# Do you think medicines can be prescribed in a more eco-directed, greener way? A qualitative study based on public and prescriber focus groups on the impact of pharmaceuticals in Scotland’s water environment

**DOI:** 10.1136/bmjopen-2024-088066

**Published:** 2025-01-20

**Authors:** Lydia Niemi, Claire Anderson, Naoko Arakawa, Mark Taggart, Stuart Gibb, Sharon Pfleger

**Affiliations:** 1 Environmental Research Institute, University of the Highlands and Islands, Thurso, UK; 2 School of Pharmacy, University of Nottingham, Nottingham, UK; 3 NHS Highland, Inverness, UK

**Keywords:** Awareness, Behavior, Decision Making, Drug Utilization, Medicine, QUALITATIVE RESEARCH

## Abstract

**Abstract:**

**Objectives:**

This qualitative study explored public and prescriber awareness of pharmaceutical pollution in the water environment and eco-directed sustainable prescribing (EDSP) as a mitigation strategy to reduce the environmental impact of prescribing in Scotland.

**Design:**

Focus groups explored prescriber and public perceptions of the topic. Common questions were asked through semistructured facilitation. Focus groups were digitally recorded and transcribed verbatim using an artificial intelligence system, then anonymised and thematically analysed using NVivo software. Data were iteratively analysed using the one sheet of paper technique.

**Setting:**

Public focus groups were held in-person (Inverness, Scotland, April 2023), and prescriber focus groups were held virtually (MS Teams, August 2023).

**Participants:**

Nine public representatives and 17 NHS Scotland prescribers participated in one of four focus groups. Purposive and opportunistic sampling approaches were used to recruit participants through social media and other channels (ie, community groups, professional emails, general practitioner and hospital flyers). Prescriber representatives registered interest through an online survey to gather information about their professional background. Responses were reviewed to ensure representation of a mixture of medical backgrounds, experience, sectors and health boards.

**Results:**

There is growing awareness among the public and healthcare professionals of pharmaceutical pollution in the environment, but further education is required on the drivers, potential effects and possible interventions. Suggestions for more sustainable healthcare included public health awareness campaigns, better provision for pharmacy take-back schemes, clear medicine/packaging labelling, regular medicines reviews and more considered patient-centred care. From the prescriber perspective, EDSP resonated well with current sustainability initiatives (eg, Realistic Medicine, switching to dry-powder inhalers), but barriers to EDSP included lack of knowledge, confidence, time and resources to implement changes. Although the public representatives were generally open to the concept of EDSP, this decision required weighing pros/cons considering personal health choices, information accessibility and transparency, and trust in and time with prescribers.

**Conclusions:**

This study identified new insights from prescribers and the public related to the concept of, and barriers to, EDSP in Scotland, as well as perspectives regarding knowledge support tools and information communication. Cross-sector and transdisciplinary collaborative approaches are needed to address the challenges identified here. Nonetheless, EDSP merits further exploration in developing more sustainable, appropriate and effective healthcare which contributes to improved public and planetary health.

**STRENGTHS AND LIMITATIONS OF THIS STUDY**

- Focus groups were used to assess public and prescriber perceptions towards the importance and feasibility of eco-directed sustainable prescribing (EDSP) to support more environmentally informed healthcare.
- A strength of the study was the paired and comparative approach which considered the public (patient voice) and prescriber viewpoints towards EDSP in Scotland.
- Transdisciplinary methods and data were integrated to develop the study design, including environmental science, qualitative health services research, prescribing and public health.
- A limitation may be the small focus group size; however, discussions were interactive, and the discourse between participants was more insightful than for individual interviews.

## Introduction

 Medicines (pharmaceuticals) are the most widely deployed healthcare intervention to diagnose, prevent, treat or cure disease and are critically important to the health and well-being of modern society. Medicine use is rising globally due to growing and ageing populations, increasing prevalence of chronic and non-communicable diseases, novel medical advances, availability and the ‘pill for every ill’ culture.^1 2^ Climate change is acknowledged as an additional driver for the increasing incidence of disease and medicine use.^3^ As climate change effects become more prevalent, health and healthcare will continue to be increasingly impacted. In 2021, the UK, and more than 50 countries signed the first Health Programme agreement at the 26^th^ United Nations Climate Change Conference of the Parties, which outlined two commitments to develop (1) climate resilient health systems and (2) sustainable, low carbon health systems.^4 5^ The UK National Health Service (NHS) boards have developed new sustainability strategies pledging to net-zero carbon emissions by 2040 and 2045.^6 7^ Much focus has been on addressing carbon emissions, with little acknowledgement of the environmental impact of healthcare on water through pharmaceutical pollution—an internationally recognised public health and environmental issue.

It is well evidenced that human-use pharmaceuticals contribute to global chemical pollution in the water environment.^8 9^ In Europe, this pollution occurs primarily via urban wastewater, following human use and disposal of medicines.^10^ However, manufacturing practices, combined sewer overflows and diffuse routes (eg, septic tanks, land-applied sewage sludge) also significantly contribute to this in some locations.^9^ Following administration (oral, intravenous, topical), pharmaceuticals are metabolised and excreted, or washed off, into urban wastewater. It is estimated that between 30% and 100% of an orally administered medicine is excreted in an unmetabolised or partially metabolised form into wastewater.^10^ Improper disposal also adds to this, with a recent study demonstrating that 35% of surveyed British people (n=230) disposed of medicines down the toilet or sink.^11^ Municipal wastewater treatment cannot fully eliminate pharmaceuticals (ie, parent compounds, metabolites and transformation products) from wastewater, resulting in a diverse mixture entering surface waters (rivers, lakes/lochs, estuaries, seas) with wastewater effluents.^12 13^ More than 630 compounds from classes including antibiotics, anti-inflammatories, antidiabetic drugs and antidepressants have been widely detected in surface waters globally.^8^ Recently, a study of 258 rivers from >100 countries were found to have 25% of sites exceeding environmental thresholds where ecotoxicological effects or selection towards antimicrobial resistance (AMR) would be expected.^14^ Pharmaceuticals are of ecotoxicological concern due to their inherent pharmacological activity which may negatively affect non-target organisms at trace concentrations (ie, levels expected in aquatic environments). Reported effects include feminisation and reproductive failure of male fish due to exposure to synthetic oestrogen hormones^15 16^ and physiological and behavioural changes in other aquatic species due to exposure to psychoactive medicines.^17^,^19^ In addition, the presence of antimicrobials in aquatic environments may promote the development and spread of AMR, a critical public health concern which is forecast to result in 10 million deaths per year and cost $100 trillion worldwide by 2050 due to the rise in drug-resistant infections.^20 21^ The direct potential risks to human health through environmental exposure to pharmaceutical pollution remain unclear. But as the close interconnection and interdependence between human-environmental-animal health becomes more apparent, it is likely that the impact of pharmaceutical pollution on water quality, ecosystem services, ecological health and biodiversity may indirectly contribute to worsening public health.

Healthcare sustainability targets in the UK call for improvements, as current prescribing practices are economically, clinically and environmentally unsustainable.^6 22^ The NHS spends >£20 billion/yr on medicines, but unused or partially used medicines are estimated to cost ~£300 million, representing a significant waste that is symptomatic of the wider imprudent administration of healthcare.^22 23^ Additionally, medicines (manufacture, transport and use) significantly contribute to the healthcare sector’s carbon footprint, accounting for ~25% of NHS carbon emissions in England.^7^ To meet sustainability targets and adapt to the triple planetary crises of climate change, biodiversity loss and pollution,^24^ the healthcare sector will need to undergo significant change in prescribing practices, in cooperation with stakeholders, including the environment and water sectors, pharmaceutical industry and the public. ‘Up-stream’ mitigation practices (eg, improving medicine selection, use and disposal), whole-systems thinking and collaboration across sectors can generate innovative, sustainable solutions which reduce pharmaceutical pollution and generate positive health impacts.^4^ Eco-directed sustainable prescribing (EDSP) is an ‘up-stream’ approach which could support development of more appropriate and effective healthcare which reduces the environmental occurrence of medicines, coupled with co-benefits in reduced costs and carbon footprints.^2 4 25^ EDSP combines two prescription optimisation methods—low-dose prescribing and rational prescribing of pharmaceuticals with reduced environmental impact profiles.^23^ Through appropriate rational prescribing to avoid pharmaceutical overuse and misuse, and prescribing pharmaceuticals with lower environmental impact based on individual characteristics (eg, ecotoxicity, biodegradability, excretion profiles), EDSP may reduce pharmaceutical pollution and create more sustainable practices.^4 23 26^ The EDSP cornerstones are to educate prescribers and patients on the environmental impact of healthcare practices and treatment options, and to provide both prescribers and patients with the correct tools and knowledge to support evidence-based and appropriate decision-making.

This study aimed to assess public (as the patient voice) and prescriber perceptions of pharmaceutical pollution in the water environment and EDSP as a potential mitigation strategy to reduce the environmental impacts of prescribing in Scotland. The objectives were to assess: (i) awareness of pharmaceutical pollution in the water environment; (ii) attitudes towards and suggestions regarding EDSP; and (iii) methods of information provision and communication. This research was undertaken as part of a project to develop novel frameworks to collate and present environmental information on selected pharmaceuticals for use as a knowledge support tool to aid healthcare decision-making.

## Methods

### Study design and participants

Qualitative focus group interviews explored public and prescriber perspectives of pharmaceutical pollution in the water environment and sustainable prescribing approaches. The study was performed by four researchers representing expertise in pharmaceutical pollution and sustainable healthcare (SP, LN) and focus group facilitation (CA, NA). Nine public and 17 NHS Scotland prescriber representatives participated in four focus group interviews in April–August 2023. Numbers were determined based on interest and availability of both public and prescriber participants, with three as the minimum number of participants per focus group, as reported elsewhere.^27^ Two in-person focus groups were held with members of the public in Inverness, Scotland, and two focus groups were held virtually (MS Teams) with NHS Scotland prescribers. Two focus groups per participant category were deemed adequate for a comparative approach and aligned with the time and resource limitations of this study. Prescriber focus groups were held virtually to reduce pressure on healthcare professionals, considering the time commitment and travel requirements. Purposive and opportunistic sampling approaches were used to recruit participants through social media and other channels (ie, community groups, professional emails, general practitioner (GP) and hospital flyers). Prescriber representatives registered interest through an online survey (Qualtrics) to gather information about their professional background (online supplemental material). Responses were reviewed to ensure representation of a mixture of medical backgrounds, experience, sectors and health boards. Researchers acted as gatekeepers organising the focus groups. Participants who indicated interest were approached via email and sent the Participant Information Sheet detailing the research study background, focus group aims and methods, expected time commitment and consent form. If willing and able, participants were then invited to one of the focus groups.

### Data collection

The project researcher (CA) conducted the focus groups, each lasting approximately 2–3 hours. Modified grounded theory was applied due to limited knowledge about this topic.^28^ Both the prescriber and public focus groups followed the same format. A short presentation was given on the background and significance of pharmaceutical pollution in the environment, the purpose of the study, and then a wide-ranging discussion was facilitated. A range of common questions were asked (online supplemental material), and the semistructured nature of the interviews ensured that all participants had the opportunity to fully reflect on their experiences and contribute to the discussion. The questions were informed by literature, including surveys among the general public and healthcare professionals on pharmaceuticals in the environment and EDSP reviews.^1026 29^,^31^

### Data analysis

Interviews were digitally recorded and transcribed verbatim using automated transcription (Microsoft Azure) for public in-person interviews, and the MS Teams transcription function for the prescriber focus groups. The anonymised data was entered into NVivo software and then thematically analysed using constant comparison.^32^ Data were iteratively analysed by two researchers, and key themes were identified using the one sheet of paper technique.^33^ Illustrative quotes were selected and agreed with the co-researchers.

### Patient and public involvement

None.

## Results

### Participant characteristics

For the two focus groups with members of the public in Inverness, six people attended one and three the other. For the two virtual focus groups with NHS Scotland prescribers, 24 of 63 NHS Scotland prescribers who completed the registration survey were invited to the focus groups to ensure a diverse mixture of participants. Due to availability, eight people attended one and nine the other. The demographics of the participants are outlined in table 1.

**Table 1:** Demographics of the public and prescriber representatives

	Public representatives	Prescriber representatives
Sex		
Male	3	10
Female	6	7
Total	**9**	**17**
Age, years		
>60	1	2
40–60	5	8
<40	3	7
Total	**9**	**17**

NHS Scotland prescribers with a range of professional backgrounds, years of prescribing experience, and from a variety of sectors and health boards were recruited into each of the two prescriber focus groups. The participant characteristics are shown in table 2.

**Table 2:** Characteristics of National Health Service (NHS) Scotland prescriber representatives from the two focus groups

Focus group	1	2
Prescribing experience, yr		
<1	1	2
1–5	1	2
6–10	5	1
>10	1	4
Professional background		
Medical doctor	1	2
Nurse	4	4
Pharmacist	3	2
Physiotherapist	0	1
Sector		
Community	0	2
Hospital	3	4
General practice	1	2
Primary care	1	1
Social care	1	0
Mixed	2	0
NHS Scotland Health Board		
Highland	1	3
Greater Glasgow & Clyde	1	0
Lanarkshire	1	2
Dumfries & Galloway	2	0
Ayrshire & Arran	2	2
Golden Jubilee	1	0
Lothian	0	1
Forth Valley	0	1

### Focus groups analysis

The following subsections highlight the key themes emerging from the focus groups. Comparative analysis of the prescriber and public focus groups revealed five common themes. Table 3 summarises the key results for both participant groups, highlighting shared views and group-specific challenges identified through the paired approach.

**Table 3:** Comparison of five common themes from both public and prescriber focus groups

Theme	Public focus groups	Prescriber focus groups
Awareness and education	Awareness of some of the issues around pharmaceutical pollution, particularly by wild water swimmers More education is needed starting at school, and awareness through GP and pharmacy posters/displays	Limited knowledge about pharmaceutical pollution, but a willingness to learn more and act More education is needed—the issue should be embedded into healthcare curricula and professional development
Information provision and messaging	Simple, clear messaging is needed, for example, labelling on medicines packaging to alert about the environmental impact and appropriate disposal Information should be provided at point of prescribing and dispensing of medicines	Accessible, reliable information is needed to aid prescribing decisions, for example, in prescribing updates and formularies Policymakers and professional leadership bodies play an important role in developing guidance and disseminating information
Eco-directed formulary	A good approach, but requires available, complete and robust data Questions raised: can accurate comparisons be made between medicines?	Supportive of an environmental section in prescribing formularies and updates, for example, in the British National Formulary and local guidance Electronic prescribing systems could aid decision-making, for example, to highlight appropriate alternatives
Switching to a more environmentally friendly medicine	Hesitancy in switching medicines to a more environmentally friendly option, unless it was equally effective and safe Depends on the condition being treated and individual health choices and goals Prescribers need to spend more time with patients discussing treatment options and building trust Reliable information is needed for this decision	Builds on current conversations and initiatives, for example, switching from dry powder inhalers, deprescribing, health promotion and Realistic Medicine* Must approach this at the right time, involving patients for shared decision-making Must consider factors like patient history, new versus long-term medications, available alternatives and polypharmacy Robust evidence and health board support is important
Developing knowledge support tools	Tools are useful, but data should be accessible and transparent with clear guidance to inform patients and the public	Tools must be simple and underpinned by robust evidence Knowledge of how the tool operates and the data included would improve confidence in interpretation A standardised classification system is needed to assess environmental impact—similar to those, for example, high prescribing volumes, costs and antimicrobial prescribing

* Realistic Medicine in Scotland is a patient-centred approach to healthcare promoting shared decision-making, patient education, medicine stewardship, and addressing waste (among others).^52^

#### Awareness and education about the issue

The participants in the public focus groups were already aware of some of the issues around pharmaceutical pollution, particularly the wild water swimmers present (eg, wastewater effluent discharge, potential impact on bathing water quality). However, they all felt there was a need for further awareness and education for all on the drivers, potential effects and possible interventions, starting at school and incorporating this topic into the curriculum.

*Yeah. And again, that I think links back to schools and education because indeed, if you’ve got your young, enthusiastic small people coming out and saying guess what we did today, we did this and look, why don’t we? How do we do that at home? You know, that is a really good way to start that whole, yeah, being shamed by your nine year old, quite a thing*. (Public 2,2)

*If you’re going to have it through the education system you’re talking about, it has to be a policy that is going to be part of the education put into the curriculum. And so, they understand that. So, unless you get government support, it’s not going to happen*. (Public 1,1)

They felt there was a need to educate adults through public health campaigns, perhaps using displays and posters in pharmacies and public areas (eg, libraries, bus stops). Community-based groups or public interest groups like the Women’s Institute, they discussed, could be a valuable resource for disseminating information.

*So safe disposal of medicines, making sure that everyone public health campaign or something around that piece*. (Public 2,1)

Some participants noted that they have seen this issue in the media and social media, where it is highlighted in headlines and then disappears again. A public participant (1,4) suggested that researchers should be involved in developing messages that communicate environmental information.

*That’s why I think the researcher, if it comes to this environmental evaluation of drugs and the impacts and simplification from the researcher side, is extremely important that the public and the general public understands better. For example, what measures are taken to analyse certain toxicity of drugs and what effects they have?* (Public 1,4)

Although the prescribers at each focus group were interested in the issue of pharmaceutical pollution, they admitted that they knew little about it. Some had heard about it in recent presentations, and they mainly felt that it was still in its infancy as an issue and was quite alarming.

*Iguess I find it quite shocking and it just worries me in terms of like extinction of species and stuff like that. If we feminise all our fish and the effect that will have on biodiversity and stuff like that.* (Prescriber 7,2)

The prescribers wanted to do something about it though, noting healthcare has a responsibility to act.

*And really quite shocking, actually. And upsetting and worrying. And yeah, none of none of it provokes good feelings. And so I would certainly and yeah, like I said, there are like really keen to try and to help as much as possible in, in making these, you know, reducing the impact of what we're prescribing because we have got responsibility for that, I think so.* (Prescriber 3,1)

They discussed the complexity of the issue and that it was difficult to see where they could make an impact. They also thought that it was a cross-disciplinary issue involving numerous stakeholders who all need to communicate and share resources and information.

*It’s definitely under the radar from a pharmacy perspective. As a pharmacist, you know, you know that these products do go into the environment, but it’s, I was kind of thinking, why is that not a thing? Because even within pharmacy, this subject is probably pretty niche, people aren't going and talking about it all the time.* (Prescriber 5,1)

Several participants noted that the issue was not just about the patient in front of the prescriber, but also needs to be considered from a population perspective.

*What I say and it’s we all, we all often have, like an obligation to the patient in front of us or we think a population level about a group of patients, hepatitis or respiratory and a kind of public health and environment. But these are downstream problems that affect everyone, but it’s almost too big to consider*. (Prescriber 6,1)

According to those in the prescriber focus groups, prescribers and patients alike would all need to be educated.

*But I think obviously patients or the general public need to be aware more because obviously it’s prescribers we’ve not been aware of this or the true impact either. So I’m sure patients don’t, I don’t either. So hence the reason a lot of patients will put things down. You know, a toilet or put them in their bins. They don’t realise maybe the implications that of what they’re doing.* (Prescriber 7,1)

They all agreed that the environmental impact of healthcare and pharmaceutical pollution needed to be incorporated into the curriculum for prescribers. Those already in practice also needed educating perhaps via NHS Education for Scotland (NES) continuing professional development courses and NHS compulsory update modules.

*I think you know putting it into this sort of curriculum for training for non-medical prescribers and also we get prescribing updates and you know working that into the updates to raise awareness of the issue.* (Prescriber 4,1)

*Otherwise I thought were like NES modules or we’ve got quite mandatory sort of NHS modules. You’ve got to do*. (Prescriber 3,1)

#### Information provision and messaging

The public representatives stated that environmental information should be incorporated into existing information which is given out with medications.

*You’ve got your summaries of product characteristics, patient information, leaflets, BNF [British National Formulary], whatever that environmental data then going to sit somewhere else, or can it be incorporated into some of these? Documents and information resources that already set out there*. (Public 2,1)

Both public focus groups agreed that a simple red, amber, green labelling system for medicine packaging (to alert people about the environmental credentials of each medicine) would be extremely useful. It would be a simple way to get the information across as there was already so much information on medicine packaging.

*You know, some of the people there was a traffic light system of red, yellow and green. You know, that would be a good thing*. (Public 2,3)

However, they noted that some informed patients may not take their medicines for environmental reasons, as is the case for some patients when informed about potential side effects. Prescribers, they thought, should be educated and have the information available, and pharmacists could inform people about the environmental hazards and risks of medicines.

*I think a lot of patients actually look at the side effects of these things and often they then decide not to take them. They might also want to look at what the risks are. Excretion or where it’s going to go, and also on the environmental reasons, decide not to take them, which is then a huge waste of course. Yeah, I think people have a choice and people have certainly younger people getting very much more aware of their effect on the environment, and rightly so*. (Public 2,3)

They also discussed that the pharmaceutical industry needed to take responsibility in making information available.

*In terms of what big pharma can do, I think there needs to be much more information available to healthcare professionals and to the public about environmental effects and the data they have*. (Public 2,1)

The prescribers indicated a willingness to act, but difficulty in identifying how to make a meaningful impact due to the complexity of the issue and lack of information. All agreed that more education and information were needed to aid prescribing decisions which could help reduce the occurrence of pharmaceuticals in the environment.

*I don’t know what is an environmentally negative medication. Don’t know what is harmful and what is not and I don’t know how you even find that that information out*. (Prescriber 2,1)

They discussed that information could be included in prescribing updates, such as an environmental section in the formulary updates. According to the prescriber representatives, information and guidance from policymakers and national bodies like the Royal Pharmaceutical Society were also important.

*For example, when we switched, everything went to using the Royal Pharmaceutical Society’s guidance and documentation on describing—so if we were going to be changing something, I think that that’s a good way to get it out to every prescriber as a higher level thinking.* (Prescriber 8,1)

The prescriber representatives also noted that it was important to involve patients in making choices about prescribing and developing more sustainable approaches. Information should be available to all.

…*It’s having that information so that the patients themselves can then get on board and make that sort of choice with you. Otherwise, it’ll probably just sit there and end up in the toilet or return to the pharmacy.* (Prescriber 6,1)

One prescriber (5,1) noted that prescribing is done ‘in partnership with the patient’ and conversations with patients needed to reflect shifts in prescribing and be clear on expectations.

*There’s almost different jobs that people have to do and obviously if we’re prescribing, we’re prescribing in like partnership with the patient, they should be aware of the goals of prescribing and the development of sustainability of what we prescribe should be part of that.* (Prescriber 5,1)

#### Eco-directed formulary

The public representatives felt that an environmentally directed formulary was a good way to make an impact. The pharmaceutical industry must perform environmental risk assessments for all new medicines. However, this information is not used to support prescribing decisions. There was some discussion on the availability, completeness and robustness of environmental data, for example, if the same level of information is available to enable accurate comparisons between medicines.

…*Create a list of good to bad*. (Public 1,1)

*Whereas an individual GP that’s like oh you know what I’d rather go for the cheaper one that’s going to work rather than eco-friendly one, because again if they’re patient-centred that’s what they’re going to look at. There may be some doctors that like do you know what, it’s cheaper, it’s good for the environment but it might not work for the patient. I’m going to try anyway, just in case. They’re not all going to be trained and the environmental impacts of these things as extra training, extra resources and teaching them.* (Public 1,5)

Information regarding environmental risk is often not available for older medicines, and this would need to be clarified to patients by prescribers.

*It does sound like a good idea. I would have thought the information would be rather patchy at the moment. So, whereas you might have good information for one drug and not so much for another, does it mean there’s better drugs or that you just don’t know yet? And I think that would be really important to make clear so that we’re not just choosing this one because it doesn’t have any bad stuff*. (Public 2,1)

The prescriber representatives discussed an environmentally directed formulary and agreed that if prescribing was to become more environmentally informed, systems such as local formularies and decision support tools are needed to be in place on electronic prescribing systems. The British National Formulary (BNF) was considered to be an important resource which could provide this information, as it was widely used not only in the UK but globally.

*And not only should we have the uses and the contraindications etcetera, you should have an environmental section on that where you can look up easily accessible and it’s a tool. Everybody uses every day and then of course you have all your local apps and whatever local formulary use, but it should be easy enough to put it onto BNF probably*. (Prescriber 9,1)

If a medicine was determined to have an unacceptable environmental risk, they also discussed the need for a tool that would then highlight the alternatives at the point of prescribing. Again, a simple traffic light red, amber, green system was suggested by the focus groups. However, it was observed that even this may cause confusion and that maybe a simple yes/no or green/red flag may be better.

*You don’t want to restrict their options, so some of might think well, I’ve got patient on a red medicine. I might be I might be reluctant to choose an amber. I might be hunting for a green, and if we just made it one or the other, it might be maybe easier for me that choice. As a prescriber, I think that would be, I think my mind might explode if I was on a ward round*. (Prescriber 5,1)

Polypharmacy made this more complicated.

*Only I was just thinking what about polypharmacy? Because patients are not just on one medication, so if you know most a lot of elderly patients are on five or six different types of medication, how are you going to, you know, they come becomes really complicated then because if one is a green or one is a yes and another one is a no.* (Prescriber 2,1)

#### Switching to a more environmentally friendly medicine

The public representatives were given the following hypothetical scenario: ‘Imagine you've been using a medicine with a potentially negative environmental effect, and you were offered the opportunity by your healthcare provider to switch to a different medicine with a lower environmental impact. Would you be willing to do that?’. The public representatives were adamant that they would not want their prescription changed to a less effective but more environmentally friendly one if that was an option. If it had the same effectiveness, most would gladly change, but this may be dependent on the condition.

*I want to be assured that it was going to be equally effective, equally as equally or even more safe than what I’d already been using. I may have to make an assessment myself as to which element of that is most important. Is it the efficacy? Is it the safety? Is it the environmental? I suppose that depends very much on what is this condition I have that I'm trying to treat and how well is that medicine I’ve been taking, been doing to help that and if it’s been brilliant then maybe I can take a little reduction in efficacy*. (Public 2,2)

*I think personally making a change in the medication if my doctors said to me, look you don’t bend the facts and but there’s other medication that’s got the exact in side effects, exact same interactions with other medications. Exactly. Medication, just different name, you know, would change. I would probably consider it*. (Public 1,5)

They discussed that it was all about weighing up the pros and cons, and patients would require information to help with this decision.

*And if I don’t like side effects, I’d go back, you know, and it’s, it’s one of those things where putting it into the patient’s hands but also giving them all the information you can in that short amount of time you have with them, you know, explaining to them the exact same medications on paper.* (Public 1,5)

*Certainly make you aware of another reason. You know if you're switching between simvastatin or atorvastatin whatever. Yeah. For example, if one had a green label and the other one had a red one, then yeah, you know, it would swing you*. (Public 2,3)

However, the participants also noted that any decision would be influenced by the person’s relationship with and trust in their prescriber. A participant related it to their GP swapping from a branded inhaler to a generic inhaler and noted that they were not prepared to make that switch on the basis of cost as they had a medication that worked well for them. Some of the participants would prefer that someone else made certain decisions for them, noting patients may not know what to do and the pressure may be too much to contend with. But there was some concern about who was making these decisions for the prescriber, and several participants noted that evidence-based decision-making was key.

*If I’m assured that they’re doing that on the basis of a complete and reliable data, yes and evidence-based decision*. (Public 2,1)

For many, this decision would need to be underpinned by the relationship with the prescriber and a mutual understanding of the condition. The patient would need confidence that the prescriber would only consider switching medicines based on environmental effects if it was appropriate for the patient.

*Yeah, I think if you’re more confident in those people then you’re more able to make those kind of decisions.* (Public 1,6)

*It’s also down to the clinician, whether it’s the doctor or anybody else understanding their patient because there have been times in my life where if somebody had given me a choice, I would have just not known what to do. Your clinician who is attending to you needs to understand when they can give you that choice and when it’s the time to see. I’m moving you to this because this is what you need, because the patient is incapable quite often of not all the time, but it’s quite often incapable of making the choice for themselves because it’s too much pressure*. (Public 1,3)

The prescriber representatives also discussed the following hypothetical situation: ‘You have been prescribing a patient a medicine with a potentially negative environmental effect. You have the opportunity to prescribe the patient a different medicine with a lower environmental impact’. The participants likened this to conversations they had already been having with patients about switching to dry powder inhalers and suggested it fed nicely into discussions around health promotion and Realistic Medicine in Scotland. Additionally, they felt that it would depend on available alternatives and how the patient was doing on the medicine, noting that it may be easier when starting new medicines rather than switching from established prescriptions. It would also be easier if a patient was already interested in environmental issues, noting that environmentally aware patients are more likely to make changes to reduce their environmental impact.

*I think the thing that patients you know they are interested in the environment and when you have the discussion and they want it to be as easy to use as the one they’ve already got*. (Prescriber 3,2)

One participant (Prescriber 8,1) noted that if they had the backing of robust evidence and could say that this switch was supported by the health board or the Scottish Government, it would also help.

*I think we’ve already sort of got that evidence for inhalers and we’re getting much better at having that conversation. So, we’re already doing it. It’s just building on what we’ve already started to say.* (Prescriber 6,1)

*You can easily switch them around, but if the patient was getting on really well with the medication and I'm I might be less willing to change them over and I think it'd be more like people always prescribing new medications too*. (Prescriber 3,1)

One participant (Prescriber 5,1) noted that primary care teams may have more opportunity to consider environmental impact and long-term patient health outcomes, rather than acute care teams. It required choosing the right time to approach the subject, and other factors such as patient history, dosage, efficacy and polypharmacy must be considered.

*Like if they had a really hard time, it’s probably it might not be the best time to kind of have that conversation with the patient. So I think it depends as well and what you’re treating the patient for, how they are as a whole and kind of mentally as well because I think that can totally change a patient’s perspective on things obviously as well.* (Prescriber 7,2)

*You know, they would be less keen to change to one that is has a lower environmental impact if for instance they had to go from taking something once a day to taking something four times a day or if it was less effective for them*. (Prescriber 3,2)

They advocated for small changes, even changing one of a patient’s medicines may make a difference.

*But I guess you know any change is better than nothing. So if you changed one, if you were able to change one medication. To have better an environmentally and better medication, and that would be a bit of a win. You wouldn't necessarily need to make everything 100% perfect, so just like small steps*. (3,1)

#### Developing knowledge support tools

As part of this research, a framework was developed to collate and model environmental hazard and exposure data to predict the environmental impact of selected pharmaceuticals in freshwater bodies in Scotland.^34^ The prescriber representatives were shown the framework structure, and data as an example of how environmental information could be used to inform healthcare decision-making in Scotland. The participants felt that while they needed to have some basic understanding of what was behind the model (ie, the data used to calculate the environmental impact scores), it needed to be very simple at the front-end, so it could be interpreted with confidence and underpinned by robust evidence. It was felt that a classification system to present environmental impact should be developed, which could identify outliers using a similar system to that currently used to identify high prescribing volumes and costs.

…*About environmental impact assessment that we need to develop a standardised methodology to assess the environmental impact of medicines…and this could involve assigning environmental impact scores. Impact score is based on factors such as chemical composition, potential for bioaccumulation, degradation rates, and known ecological effects…it’s about training health care professionals about the environmental impact of medicines and how to interpret those environmental impact scores. And this then would enable them to have informed conversations with patients about the ecological consequences of different medications.* (Prescriber 2,2)

They discussed that changes in prescribing could then be communicated following processes already in place. Antimicrobial prescribing reports (for example) are used to compare performance towards prescribing targets between practices.

*I think it’s quite complex. You’ve got things like some of the quarterly antimicrobial steward reports which show practices are clusters, practices are performing in comparison to like other practices within the local health board.* (Prescriber 6,1)

The prescribers were also shown how environmental impact could be visualised and communicated through a Scotland-wide map which categorised geographical areas based on the likelihood of a pharmaceutical exceeding the threshold level where ecotoxicological effects are expected in freshwater. They felt a map of Scotland with levels of water pollution was useful but also had its limitations. For example, several participants discussed the difficulty in attributing direct outcome measures based on medicines when so many other factors were involved (eg, population demographics, prescribing trends, multiple pollutants, environmental conditions).

…*It must be very difficult to pick an outcome measure in the environment and then isolate the impacts of pharmacology on that because I've obviously there’s other impacts such as what we talked about, I don't know, is it next to an oil plant or something like that? And it’s picking the right outcome measures that can be monitored that we know pharmacology’s impacting directly and then seeing over time is that improving with altered practice*. (Prescriber 2,2)

Additionally, local guidance may be more appropriate, rather than Scotland-wide, due to location-specific or compound-specific conditions affecting how prescribers and patients should respond. There may be an opportunity to observe changes in the environmental impact over time with altered prescribing activities.

*With the maps it you know, presumably because you know there’s less people in the Highlands, you know, I was looking at that map about where I live and then some things that might have an impact in Glasgow won't have an impact with me.* (Prescriber 3,2)

Other options (regarding environmentally friendly prescribing) were suggested by both groups, including reducing the number of medicines prescribed per day (if appropriate), short courses for new medicines, regular medicine reviews to avoid unnecessary repeat prescriptions, deprescribing and social prescribing. While acknowledging the pressures on healthcare staff, the public representatives agreed that prescribers need to spend more time with patients discussing treatment options, including those other than medicine.

*So we use ecotoxic medicines frequently, and most patients are commenced on methotrexate as their first line kind of treatment. So I’m very interested in how this has an impact on the environment and how we can obviously be helpful in that. We do try and give patients short prescriptions and try and reduce waste that way, but obviously I’d never thought about it*. (Prescriber 7,1)

*So our prescriptions at the moment are set up when you prescribe, you probably just prescribe a month’s worth and because you know that’s often if you’re trying a new thing, what you’ll do, whereas actually if we wanted to reduce waste, if you had a system where you could prescribe a week and then if you’re OK after the week, you pick up the other three weeks from the chemist*. (Prescriber 3,2)

Whatever was done had to be easy and quick to do in a busy clinic.

*Because people will do things that are easy, but if they’re difficult, there’s another barrier there and even though they might want to do the right thing and do the thing that is green and sustainable and better for the environment, if it’s not easy, they're not going to do it.* (Prescriber 3,2)

## Discussion

### Summary

This study provides insights into public and prescriber perspectives in Scotland towards pharmaceutical pollution in the water environment and EDSP as a potential mitigation strategy to support more sustainable and effective healthcare practices. From the public perception, while participants were aware of some of the issues related to pharmaceutical pollution, they all felt there was a need for further education. This could start with educating school-aged children, using health promotion methods such as pharmacy displays, or through better medicine labelling to alert people about appropriate disposal and the environmental impact of medicines. It was agreed that better provision for disposal of unwanted medicines was needed through more accessible pharmacy return schemes, particularly in rural areas. Additionally, more considered patient-centred care was needed, as was a greater focus on regular medicine reviews and deprescribing. They felt prescribers needed to spend more time with patients discussing treatment options, including those other than medicines. Public participants were also adamant that they would not want their prescriptions changed to be less effective but more environmentally friendly (if that was an option). If effectiveness were equal, they would gladly change, but this required weighing up the pros/cons, discussions with prescribers, and clear and accessible information to help decision-making.

From the prescriber perspective, participants all agreed that pharmaceutical pollution needed to be incorporated into training. Prescribers generally demonstrated a willingness to enact more sustainable prescribing but acknowledged the complexity of the issue and the challenge in making a meaningful impact through individual actions. When discussing switching a patient to a more environmentally friendly medicine, it was noted that similar conversations already occurred with patients about certain changes (eg, for inhalers), and EDSP would align with this. They also discussed the cross-disciplinary nature of this issue and the need to share and communicate information across sectors in a clear and simple way. There was agreement on the need for accessible information, such as environmental sections in formularies and decision support tools which could be integrated into electronic prescribing systems. Other opportunities to drive more sustainable practices included regular prescription reviews, short courses, and promoting deprescribing and social prescribing (where appropriate). All participants stressed the importance of involving patients in decisions about environmentally informed prescribing, emphasising the need for more education and awareness, as well as clear labelling and disposal instructions.

### Strengths and limitations

The strength of this study was in its paired and comparative approach which considered the public (patient voice) and prescriber viewpoints towards EDSP in Scotland. This study provides preliminary insights into the perceptions of these two groups towards the importance and feasibility of sustainable prescribing initiatives, through exploring awareness and attitudes towards pharmaceutical pollution in the environment and novel approaches which could support more environmentally informed healthcare in future. This was a small in-depth qualitative study—the public came from one area of Scotland and the prescribers from across Scotland. As such, it was never meant to be generalisable to other settings, although findings in other settings may be similar. A limitation may be the small focus group sizes; however, discussions were interactive, and the discourse between participants was more insightful than for individual interviews. Other studies have also found that small groups (ie, of three) can result in valid focus group interview data.^27^ Additionally, it is possible that prescribers with pre-existing awareness and interest on pharmaceutical pollution and sustainability may have chosen to participate in the focus groups. The facilitators did not seek to recruit prescribers based on their interest in healthcare sustainability or eco-directed prescribing, and those who registered interest were not asked to clarify this in the recruitment survey. Despite this potential bias, the researchers believe that the findings are still valuable to inform next stage research. Furthermore, only one data type was collected (focus groups) at one time point, and collecting multiple types of data or longitudinal data (ie, multiple focus groups with the same participants at different time points) could bolster the theoretical knowledge gained. Nonetheless, the authors are confident in the merit of the research, which led to important insights into how prescribing practices in Scotland can become more environmentally informed.

### Comparison with literature

#### Awareness and education

Both public and prescriber representatives were aware of the issue of pharmaceutical pollution in the water environment, but further awareness and education were needed. Both groups acknowledged the important role of the public in mitigating the presence of pharmaceuticals in the environment. In a study in Finland, it was also clear that the public was increasingly aware of the presence of pharmaceuticals in the water environment, but the primary pathways and role of individuals in contributing to this issue were less understood.^10^ Additionally, when compared with environmental pollution from agricultural practices and veterinary medicines, human-use pharmaceuticals were generally considered (by the public) as less severe.^30 35^ There may also be assumptions that over-the-counter medicines (eg, anti-inflammatories, pain killers) are less hazardous than prescription medications such as antibiotics, hormones and antidepressants.^36 37^ As such, directed efforts (by medicine-type or condition) through disposal instructions on medicine packaging or patient information leaflets, with verbal instructions at the point of prescribing, may be effective in influencing behaviour.^30 38^ The media may also have an important role to play (in raising awareness and influencing behaviour), but this must be sensitive to target groups and avoid a ‘one-size-fits-all’ assumption to ensure that information is comprehensive and accessible to target groups.^37^ Both communication credibility and its impact on pharmaceutical pollution could be improved by engaging government authorities and professional leadership bodies in oversight and dissemination. This would require further cooperation between key relevant stakeholders (ie, public health, pharmaceutical, healthcare, water and environmental sectors).^39^ Here, it was suggested that pharmacy displays and media campaigns covering the associated public health aspects of pharmaceutical pollution could be effective to inform appropriate action.

The prescriber focus groups here suggested that incorporating pharmaceutical pollution and the environmental aspects of healthcare into teaching curricula was necessary to better educate and normalise this issue among healthcare professionals. One study found that students were generally positive about incorporating this issue into curricula.^40^ When considering practising prescribers, although physician respondents to a survey showed a willingness to apply EDSP practices, no respondents indicated that the environmental impact of a medicine would affect their prescribing decisions due to limited knowledge and confidence.^26^ Interdisciplinary training has however been successfully applied to educate clinicians on pharmaceutical pollution.^37^ Integrating environmental aspects into educational materials may improve prescriber awareness and support their confidence in following any guidance with action. It is however important that training strategies include opportunities for discussion regarding the feasibility of applying learning into practice and how actions could be incorporated into day-to-day work.^37^ Here, it was suggested that oversight and facilitation is needed by bodies such as the British Medical Association, and professional leadership bodies could likely have an additional role to play (eg, the Royal Pharmaceutical Society, Royal College of General Practitioners).

#### Knowledge support and eco-directed formulary

Although healthcare professionals may be increasingly aware of the environmental impact of prescribing practices, this infrequently translates into action. The main barriers to implementing more environmentally informed prescribing include limited knowledge of how to change practice, lack of time and resources to facilitate changes, ethical concerns and a lack of policy underpinning these decisions.^26 31 41 42^ One study investigating this topic with NHS England hospital pharmacists found that participants did not believe that environmental considerations were part of their job.^43^ Here, the public and prescriber focus groups were generally supportive of an eco-directed formulary or the inclusion of environmental information into existing resources like the BNF. Examples exist in Sweden, where publicly available databases present environmental information of pharmaceuticals on the Swedish market for consideration in prescribing decisions. The Drug and Therapeutics Committee (DTC) in Stockholm collates and summarises environmental risk and hazard data of pharmaceuticals on the *Pharmaceuticals and Environment* database (www.Janusinfo.se)—which aims to support more environmentally-informed prescribing, following clinical and cost considerations and subject to data completeness.^39 44^ Janusinfo includes information from the pharmaceutical industry, available through the Fass database (www.fass.se), developed by the Swedish trade association for the research-based pharmaceutical industry (LIF).^39 45^ Databases like these may support knowledge transfer, but mechanisms are needed which facilitate simple, clear and time-effective interpretation and use of environmental information for healthcare professionals.^26 31^ Here, the prescribers were presented with a framework to predict the environmental impact of a selected pharmaceutical in the Scottish freshwater environment, by modelling and mapping environmental hazard and exposure data across different geographical areas in Scotland. The framework and map were attractive to prescribers as a method to condense and communicate complex information in the Scottish context, building on databases which present information. As reported elsewhere, healthcare practitioners may be unable to critically assess and interpret the individual environmental factors used to determine environmental risk and hazard.^31^ To facilitate simple access, this framework could sit within existing electronic prescribing interfaces to communicate environmental impact to healthcare professionals (eg, within ScriptSwitch, e-formulary). Prescribers also suggested that further refinement could consider more granularity at the spatial scale to inform local formulary development and prescribing guidance (eg, as for Antibiotics Awareness and Antimicrobial Stewardship).

However, providing information alone is not enough to change practice, and the lack of healthcare regulatory guidance and policy is a major challenge.^41 46 47^ In the UK, there is currently no standardised framework that integrates environmental information into healthcare decision-making processes (ie, market authorisation, health technology assessment (HTA), formulary, prescribing).^46 47^ The Medicines and Healthcare Products Regulatory Agency regulates pharmaceuticals manufactured domestically and imported, and although environmental risk information is required, this is not currently considered during market authorisation.^46^ Further, environmental information is not used by the Scottish Medicines Consortium when conducting HTAs to inform local formulary decision-making in Scotland.^48^ Formulary subgroups of Area Drug and Therapeutics Committees consider efficacy, safety and cost-effectiveness in formulary recommendations, and again environmental information is not routinely available or considered. Swedish DTCs identified difficulties with weighting environmental impact against efficacy and cost-effectiveness, stressing the need for regulatory oversight and legislation to include environmental risk in the overall risk-benefit analysis during market authorisation.^31^ This is further complicated since environmental risk assessments (ERAs) on individual pharmaceuticals did not become mandatory 2006 until (for market access applications of new medicines). The European Commission’s Strategic Approach to Pharmaceuticals in the Environment called for mandatory ERAs following European Medicines Agency guidelines, enforcement of risk mitigation measures, and increasing data transparency, among others.^49^ However, the lack of comprehensive ERAs has affected decision-making towards risk mitigation and changes in clinical practice, such as the use of environmental information in healthcare decision-making in the UK.^4 47^ A recent policy review investigated future perspectives on EDSP in Scotland and recommended that current obstacles should not limit the development of proof-of-concept and user-friendly environmentally informed knowledge support tools which could be used by healthcare decision-makers.^46^

#### Switching to a more environmentally friendly medicine

Regarding the hypothetical scenarios about switching medicine use/prescription to a more environmentally friendly medicine, the public representatives agreed that the choice is individual-specific. As reported elsewhere, for less severe conditions, the public were willing to consider environmentally friendly options and non-pharmacological options, when informed about environmental impact.^35 37^ However, it is often essential to use medicines, and frequently environmental impact is outweighed by the potential benefit of the intervention,^10 35^ although there are instances where medicine use and improved health outcome do not correlate.^41 50^ Additionally, care is needed regarding ‘regretful substitution’ where comparative assessment of a medicine and potential alternative cannot be performed due to inconclusive or inadequate environmental data (ie, eco-toxicity, metabolites).^23 25^ As such, users of medicines need appropriate, robust information to support more environmentally informed decision-making about medicine use and disposal, which will not affect adherence.^10 41^ In this study, public participants were also sensitive to the potential stigma and mental burden of using medicines which may have adverse environmental effects, particularly in treating long-term conditions. Patient involvement is therefore critical to consider the pros/cons with respect to individual choices, and shared decision-making should be underpinned by confidence and trust through a positive patient-prescriber relationship.^29 35^

Evidence-based and shared decision-making were identified as key factors influencing both the public and prescribers when considering changes in medicine.^4 41^ Many of the prescribers referenced similar conversations with patients about inhalers, as NHS Scotland and the Scottish Government progress policy and healthcare guidance to address the environmental impacts of carbon emissions from respiratory prescribing. Current advice is to switch inhalers to the less impactful dry powder inhalers, following a patient review and not through a blanket switch.^51^ Similarly, the concept of working with patients to switch medicines to environmentally greener alternatives is aligned with the Realistic Medicine approach in Scotland. Realistic Medicine supports more personalised and equitable healthcare, and provides recommendations on how to adopt green practice behaviours in healthcare (eg, fostering stewardship, promoting non-pharmacological interventions, addressing waste).^52^ It also promotes shared decision-making and patient-centred approaches through self-management, lifestyle adjustment and resilience building.^52^ The importance of patient education within treatment is highlighted, with tools and guidance for specific conditions (eg, diabetes, respiratory disease, chronic pain).^52^ This is clearly an important strategy in the Scottish policy landscape to support sustainable healthcare in Scotland. There are also behavioural aspects considering prescribing habits, and potential professional and social pressures with the ‘perceived norm’ and ‘wait and see’ perspective highlighted elsewhere.^26 53^ Identifying when is best to deploy a potential switch in medicine to a greener alternative would be based on the level of understanding by the prescriber not only of the treatment alternatives, but also of the patients and their health. EDSP could also sit well alongside clinical ‘conservative prescribing’ and personalised ‘precision’ medicine,^23 25^ where rational prescribing to control excess medicine prescription may be a good first step to improve effective, sustainable prescribing and reduce pollution.^26^

### Other considerations

Many of the alternative approaches suggested in the prescriber focus groups to address the environmental impact of healthcare and prescribing have been reported elsewhere. In the UK context, healthcare professionals identified potential interventions through medicines optimisation, patient involvement, waste and disposal, and nature-based social prescribing.^41^ Similarly, DTCs in Sweden suggested advocating more preventative solutions, and using more non-pharmacological treatments.^31^ In particular, nature-based social prescribing may contribute to both reactive (healthcare) and proactive (health promoting) public health solutions while enhancing the natural environment.^25^ Additionally, there is a call for regular medication reviews, following growing evidence that large volumes of medications are unused, as a potential result of discontinued use, over-dispensing and over-prescribing.^30^ Here, suggestions to address this included reducing the number of tablets prescribed, minimising repetitions of unnecessary medicines and promoting deprescribing—recognising that polypharmacy is an issue which makes this more complex. Low-dose prescribing may have multiple benefits including reducing environmental loads, eliminating waste from unused medicines and protecting public health from unintended poisonings from inappropriate storage/disposal.^26 30^ However, there are concerns around achieving optimal therapeutic efficacy and delayed treatment. In a survey of health professionals, scientists, environmental authorities and pharmaceutical industry and patient representatives in the UK, Germany and Hungary, standardised regulation for appropriate medicines disposal and public awareness campaigns were among the top three prioritised approaches to address the environmental occurrence of pharmaceuticals, as opposed to environmentally informed formularies.^37^ And there may be further opportunities to consider environmental impact across product life cycle, particularly for new drugs, including manufacturing, procurement and supply chains.^54 55^ This requires regulatory oversight and transparency in the product chain, but positive enablers here include the New Zealand initiative, MedSafe, which made domestic manufacturing information on pharmaceuticals publicly available.^31^

There was discussion that the pharmaceutical industry should take responsibility for the pollution which results from medical products. Initiatives such as extended producer responsibility schemes are rapidly developing in EU legislation. Revisions to the Urban Wastewater Treatment Directive include implementation of the ‘polluter pays’ principle, where the pharmaceutical and cosmetics sectors will pay 80% of costs for installation of quaternary wastewater treatment, to reduce pharmaceuticals and other priority substances entering the water environment.^56^ Additionally, efforts should be noted regarding improved transparency and accessibility of environmental data from the pharmaceutical industry. In Sweden, environmental information is publicly available per medicinal product on the Fass system, owned by the pharmaceutical industry.^45^ In the UK, AstraZeneca launched an online *Ecopharmacovigilance* database to inform on the global environmental risk of their pharmaceutical products, by mapping pharmaceutical occurrence in the water environment (from literature) and determining environmental risk.^57^ Although these voluntary initiatives could be improved through external independent review, it should be acknowledged that the pharmaceutical industry is improving in willingness and follow-through to develop publicly available systems to communicate environmental information. Other initiatives exist, such as the research-industry collaborative project ‘Prioritisation and Risk Evaluation of Medicines in the Environment’ (https://imi-premier.eu) which aims to create an open-access database and tools assessing the environmental fate and effects of pharmaceuticals with limited data availability (ie, off-patent, legacy or pre-2006 pharmaceuticals).^58^ Resources such as these are vital to fill current knowledge gaps through provision of robust, accurate and quality-assured environmental data and thus enable development of substantiated and credible knowledge support tools to communicate environmental information to healthcare decision-makers and the public.

## Conclusion

This study explored the perspectives of public and prescriber representatives in Scotland towards pharmaceutical pollution in the water environment and environmentally directed prescribing. There is growing awareness among the public and healthcare professionals regarding pharmaceutical pollution in the environment, but further education is needed for all on the drivers and potential effects of, and interventions for, this issue. Multiple recommendations were given to support more environmentally friendly healthcare, including public health awareness campaigns, better provision for pharmacy take-back schemes and appropriate disposal initiatives, clear medicine/packaging labelling, regular medicine reviews and more considered patient-centred care. Important considerations were identified regarding the impact of, and barriers to EDSP in Scotland, as well as perspectives on knowledge support and information provision to communicate environmental impact data. From the prescriber perspective, EDSP resonated well with current initiatives around switching to dry-powder inhalers and Realistic Medicine (ie, fostering stewardship, addressing waste, promoting non-pharmacological interventions and shared decision-making). But barriers included lack of knowledge, confidence, time and resources to implement changes. Although the public representatives were generally open to the concept of EDSP, this decision required weighing pros/cons in relation to personal health choices and goals, access to clear and transparent information, and trust in and time with prescribers. There is an evident need for accessible and robust knowledge support tools to enable EDSP, which should be underpinned by policy guidance and embedded into existing systems (eg, formularies, electronic prescribing software) to facilitate changes in prescribing decision-making. To establish EDSP in Scotland, collaborative and transdisciplinary efforts are needed to address challenges outlined here including prescriber and public education, data gaps (ie, availability, robustness, transparency), development of effective knowledge support tools and regulatory oversight. With growing pressure to meet sustainability targets, healthcare will need to undergo fundamental changes to create more sustainable, appropriate and effective methods which improve public and planetary health. EDSP may be one potential solution which merits further exploration.

## supplementary material

10.1136/bmjopen-2024-088066 online supplemental material 1

## Data Availability

No data are available.
