# Supplementary material for: Do you think medicines can be prescribed in a more eco-directed, greener way? A qualitative study based on public and prescriber focus groups on the impact of pharmaceuticals in Scotland’s water environment
Source: BMJ Open. 2025 Jan 20;15(1):e088066. doi: 10.1136/bmjopen-2024-088066 (PMC11749214; doi:10.1136/bmjopen-2024-088066)
Supplement: online supplemental material 1 [file bmjopen-15-1-s001.docx]

**Supplementary Material**

1. **Prescriber Registration Survey – Questions**

Q1. Do you wish to register interest to participate in the online prescriber focus group?

Q2. Which online focus group session would you prefer to participate in? You will be asked to participate in one 2 hr session.

Please indicate a date that would suit you better. We will hold the focus groups in the evening, 18:00-20:00.

Q3. Your name

Q4. Your email address

Q5. Your job title

Q6. The NHS Scotland health board which you are affiliated with (if applicable)

Q7. Your organisational affiliation if you answered "None of these" above

Q8. Your professional background

Q9. Your professional background - if you selected Non-medical prescriber

Q10. Your professional background if you answered "other" above

Q11. Your sector representation (select all that apply)

Q12. Your sector representation if you answered "other" above

Q13. Your years of experience in prescribing

Q14. Your age

Q15. Your gender

Q16. Please note any further remarks or comments for the research team to consider in the text box below.

1. **Public Focus Group – Questions**
2. What do you think about the presentation?
3. Have you previously heard about pharmaceutical pollution in the water environment?
4. Pharmaceuticals have biological effects on humans, did you know that pharmaceuticals may have similar biological effects on aquatic organisms?
5. Do you consider pharmaceutical pollution in the water environment to be an important issue?
6. Do you think medicines can be prescribed in a more eco-friendly, greener way?
7. A formulary is a list of preferred prescribing choices for clinicians. Do you think the environmental impact of medicines could be included in formularies, alongside the current criteria (e.g., patient safety and suitability, clinical effectiveness, cost)?
8. What are your thoughts on this hypothetical situation: You have been using a medicine with a potentially negative environmental effect, and you are offered the opportunity by your healthcare provider to switch to a different medicine with a lower environmental impact.
   1. Would you be willing to make this change in medicine?
   2. What information would you need to allow you to make a more environmentally directed choice on medicine use?
   3. Would you be willing to make this change in medicine, if the alternative medicine was a little less effective but was less harmful to the environment?
9. Shared-decision making and a patient-centred approach to prescribing is becoming more important in healthcare.
   1. Would you discuss the environmental impact of a medicine with your healthcare prescriber, if this was important in your health choices and goals?
   2. What do you think is the best way to engage patients in discussions which may result in the prescription of a medicine?
10. Who should be responsible for raising public awareness on the environmental impact of medicines, and how can this be done?
11. **Prescriber Focus Group – Questions**
12. What do you think about the presentation?
13. How do you feel about pharmaceutical pollution in the water environment?
14. How could medicines be prescribed in a more environmentally directed and sustainable way?
15. Researchers have collected data environmental information of selected pharmaceuticals, to determine an environmental impact score in the water environment in Scotland.
    1. How could these types of data be used to build a picture of the impact of a medicine on the aquatic environment?
    2. Do you have suggestions on how to present this information to healthcare professionals to help inform more eco-directed prescribing choices?
    3. Where should such information be used?
16. How could information on the environmental impact of medicines be included in prescription formularies, alongside the current criteria (e.g., patient safety and suitability, clinical effectiveness, cost)?
17. What are your thoughts on this hypothetical situation: You have been prescribing a patient a medicine with a potentially negative environmental effect. You have the opportunity to prescribe the patient a different medicine with a lower environmental impact.
    1. Would you be open to and willing to discuss aspects on the environmental impact of a medicine with a patient, if it was important to their health choices and goals?
    2. Would you be willing to make a change in prescribed medicine?
    3. What would you need to support this decision?
18. Do you think there are any ways we could raise prescriber awareness and knowledge on the environmental impact of pharmaceuticals?
    1. How could this come into practice?
    2. Who could be responsible for this?
19. Anything else to say?
