# Supplementary material for: Do you think medicines can be prescribed in a more eco-directed, greener way? A qualitative study based on public and prescriber focus groups on the impact of pharmaceuticals in Scotland’s water environment
Source: BMJ Open. 2025 Jan 20;15(1):e088066. doi: 10.1136/bmjopen-2024-088066 (PMC11749214; doi:10.1136/bmjopen-2024-088066)
Supplement: Supplementary file 2 [file bmjopen-2024-088066.reviewer_comments.pdf]

PEER REVIEW HISTORY

BMJ Open publishes all reviews undertaken for accepted manuscripts. Reviewers are asked to complete a checklist review form and are provided with free text boxes to elaborate on their assessment. These free text comments are reproduced below.

ARTICLE DETAILS

Title (Provisional)

Do you think medicines can be prescribed in a more eco-directed, greener way? A qualitative study based on public and prescriber focus groups on the impact of pharmaceuticals in Scotland's water environment

Authors

Niemi, Lydia; Anderson, Claire; Arakawa, Naoko; Taggart, Mark; Gibb, Stuart; Pfleger, Sharon

---

VERSION 1 - REVIEW

**Reviewer**	**1**
**Name**	**Moqbel, Shadi**
**Affiliation**	**The University of Jordan**
**Date**	**14-Aug-2024**
**COI**	**No competing interests**

Dear Editor/ Authors

The manuscript presents an interesting study. Although the study had low number of participants, the open discussion approach with semi-structured questionnaire overcome the need for higher number of participants. My only concern regarding this study is that it does not contribute significantly to the global knowledge. Most of the outcomes and recommendations are already known and described in literature. Nevertheless, It seems that the results are valuable to Scotland and the UK. Therefore, I recommend accepting this manuscript.

**Reviewer**	**2**
**Name**	**Luís, Sílvia**
**Affiliation**	**Centro de Investigação e de Intervenção Social, Iscte - Instituto Universitário de Lisboa**
**Date**	**22-Sep-2024**

COI **No**

This study analyses EDSP, a strategy that optimizes healthcare by reducing the environmental impact of medicines, as a potential mitigation approach in Scotland. The objectives—assessing awareness of pharmaceutical pollution, attitudes toward EDSP, and methods to enhance communication and information dissemination—were successfully achieved. The study effectively identified gaps in both public and prescriber knowledge regarding pharmaceutical pollution and the benefits of EDSP. This research is vital for understanding how to better promote EDSP and foster more sustainable healthcare practices.

I recommend: 1) incorporating a figure that visually summarizes the key results from the prescriber and public focus groups. This would make the findings easier to comprehend. A side-by-side comparison using columns or charts could highlight both shared views and group-specific challenges; and 2) acknowledging a limitation concerning the generalizability of the results, due to the small sample size and the single data collection point.

Reviewer **3**

Name **Singleton, Judith**

Affiliation **Queensland University of Technology, Faculty of Health, School of Clinical Sciences**

Date **30-Sep-2024**

COI **[None indicated].**

Dear authors,

Thank you for the opportunity to review this manuscript on an incredibly important global issue - the impacts of pharmaceuticals on the environment. The comparison of the consumer and the prescriber stakeholders' perspectives was also very informative.

GENERAL COMMENTS

This paper is very well written and easy to read - thank you. Commendably, the authors have taken one very small component of a larger project but tied the findings to a very comprehensive review of resources/tools/databases to inform the larger study. It was clear to this reviewer that the researchers had conducted a thorough, comprehensive literature and web review.

Well done!

DETAILED COMMENTS

INTRODUCTION

Line 52: 50 countries not counties

Line 60: sentence is too long and needs reworking

Line 66: 35% of British people - this statistic requires clarification - is it 35% of the sample British population of 230 people?

METHODS

Line 146: Charmaz reference missing from References List

DISCUSSION

This is not a revision but a general comment - "Public participants were also adamant that they would not want their prescriptions changed to be less effective but more environmentally friendly (if that was an option)." I have found the same thing in my research so while patient excretion is the main entry point we need to also continue to exert pressure for innovation (green chemistry) in the drug development stages.

Line 537: remove comma after 'here'

Line 540: practising NOT practicing

Line 553: the barriers identified here were also identified in research conducted with NHS hospital pharmacists in England ("Barriers identified to the inclusion of sustainability considerations in clinical decision-making were lack of knowledge of the environmental risk profiles for treatment options, lack of quality environmental evidence incorporated into clinical guidelines and lack of time to research for themselves. Many participants did not believe that environmental considerations were part of their job."

Singleton, J. A., Lau, E. T., & Nissen, L. M. (2022). An exploration of hospital pharmacists' engagement with sustainability policy in the NHS England. International Journal of Pharmacy Practice, 30(4), 383–390. https://doi.org/10.1093/ijpp/riac040

Line 616: perhaps begin this sentence with "In this research project ...." rather than here

Line 658-663 - this sentence is clumsy and too long - please rework.

"In other studies ...."

CONCLUSION

Line 693: "... further education is needed for all around the drivers and potential effects of.." - the wording "for all around" needs revising.

VERSION 1 - AUTHOR RESPONSE

Reviewer #1:

Dr. Shadi Moqbel, The University of Jordan
R1.1. Comments to the Author:
Dear Editor/ Authors
The manuscript presents an interesting study. Although the study had low number of participants, the open discussion approach with semi-structured questionnaire overcome the need for higher number of participants. My only concern regarding this study is that it does not contribute significantly to the global knowledge. Most of the outcomes and recommendations are already known and described in literature. Nevertheless, It seems that the results are valuable to Scotland and the UK. Therefore, I recommend accepting this manuscript.

- The authors thank the reviewer for their positive review and support of the manuscript for publication. The authors agree that although no “new” information was learned in the global context, the study performed a valuable investigation of a topic which is novel and not well understood in the healthcare sector and public spheres in Scotland. It has enabled insightful conclusions to be drawn on next steps to further engage with prescriber and public groups to overcome current knowledge gaps and barriers. This includes informing communication campaigns and educational strategies to drive action towards more appropriate, environmentally-informed use of medicines in Scotland. Considering the healthcare sustainability targets set in Scotland and the wider-UK, this will contribute to progressing the state of the art at the national and European level towards improving both public and environmental health.

Reviewer #2:

Dr. Sílvia Luís, Centro de Investigação e de Intervenção Social, Iscte - Instituto Universitário de Lisboa
Comments to the Author:
This study analyses EDSP, a strategy that optimizes healthcare by reducing the environmental impact of medicines, as a potential mitigation approach in Scotland. The objectives—assessing awareness of pharmaceutical pollution, attitudes toward EDSP, and methods to enhance communication and information dissemination—were successfully achieved. The study effectively identified gaps in both public and prescriber knowledge regarding pharmaceutical pollution and the benefits of EDSP. This research is vital for understanding how to better promote EDSP and foster more sustainable healthcare practices.

I recommend:

R2.1. 1) incorporating a figure that visually summarizes the key results from the prescriber and public focus groups. This would make the findings easier to comprehend. A side-by-side

comparison using columns or charts could highlight both shared views and group-specific challenges; and

- The authors thank the reviewer for this suggestion, and agree with the inclusion of a visual to summarise the key results and improve clarity for the reader. Table 3 presents a side-by-side comparison to highlight both shared views and group-specific challenges. See additions to lines 187-192.

R2.2. 2) acknowledging a limitation concerning the generalizability of the results, due to the small sample size and the single data collection point.

- Please refer to Discussion section "Strengths and limitations", where the limitations are highlighted. It has been updated to include the generalizability in line 524-527, stating: "This was a small in-depth qualitative study – the public came from one area of Scotland and the prescribers from across Scotland. As such it was never meant to be generalisable to other settings, although findings in other settings may be similar."

Reviewer #3:

Dr. Judith Singleton, Faculty of Health, School of Clinical Sciences (Pharmacy), Queensland University of Technology
Comments to the Author:
Dear authors,
Thank you for the opportunity to review this manuscript on an incredibly important global issue - the impacts of pharmaceuticals on the environment. The comparison of the consumer and the prescriber stakeholders' perspectives was also very informative.

GENERAL COMMENTS
This paper is very well written and easy to read - thank you. Commendably, the authors have taken one very small component of a larger project but tied the findings to a very comprehensive review of resources/tools/databases to inform the larger study. It was clear to this reviewer that the researchers had conducted a thorough, comprehensive literature and web review.
Well done!

- The authors thank the reviewer for their positive response, and support of this study. It is much appreciated!

DETAILED COMMENTS
INTRODUCTION
R3.1. Line 52: 50 countries not counties

- Amended, see line 57.

R3.2. Line 60: sentence is too long and needs reworking

- Amended, see line 64-67.

R3.3. Line 66: 35% of British people - this statistic requires clarification - is it 35% of the sample British population of 230 people?

- Amended, see line 71.

METHODS

R3.4. Line 146: Charmaz reference missing from References List

- Added to reference list – Charmaz, K. (2006). *Constructing grounded theory: A practical guide through qualitative analysis*. sage.

DISCUSSION

This is not a revision but a general comment - "Public participants were also adamant that they would not want their prescriptions changed to be less effective but more environmentally friendly (if that was an option)." I have found the same thing in my research so while patient excretion is the main entry point we need to also continue to exert pressure for innovation (green chemistry) in the drug development stages.

- The authors agree that interventions are needed across the full lifecycle of the pharmaceutical product chain, and it is likely that large-scale changes could be best instigated at the production/development stage. But with hesitancy from public/patients, the authors think there is still value in pushing for more education and awareness around medicine stewardship/rational use, healthy lifestyles, appropriate disposal, and down-stream effects. In future, all these mechanisms working together may lead to improved public health and reduced environmental pollution through more effective, appropriate, and sustainable prescription, use, and disposal of medicines.

R3.5. Line 537: remove comma after 'here'

- Amended, see line 557.

R3.6. Line 540: practising NOT practicing

- Amended, see line 560.

R3.7. Line 553: the barriers identified here were also identified in research conducted with NHS hospital pharmacists in England ("Barriers identified to the inclusion of sustainability considerations in clinical decision-making were lack of knowledge of the environmental risk profiles for treatment options, lack of quality environmental evidence incorporated into clinical guidelines and lack of time to research for themselves. Many participants did not believe that environmental considerations were part of their job."
Singleton, J. A., Lau, E. T., & Nissen, L. M. (2022). An exploration of hospital pharmacists' engagement with sustainability policy in the NHS England. International Journal of Pharmacy Practice, 30(4), 383–390. https://doi.org/10.1093/ijpp/riac040

- The authors thank the reviewer for highlighting this relevant and insightful study – it has been added to the manuscript. See lines 575-577.

R3.8. Line 616: perhaps begin this sentence with "In this research project ...." rather than here

- Amended, see line 632.

R3.9. Line 658-663 - this sentence is clumsy and too long - please rework.
"In other studies ...."

- Amended, see lines 673-675.

CONCLUSION

R3.10. Line 693: "... further education is needed for all around the drivers and potential effects of.." - the wording "for all around" needs revising.

- Amended, see line 707.

VERSION 2 - REVIEW

Reviewer 3

Name Singleton, Judith

Affiliation Queensland University of Technology, Faculty of Health, School of Clinical Sciences

Date 19-Nov-2024

COI

Dear authors,

Thank you for your comprehensive revision of the original manuscript and your comprehensive response to editorial and reviewers' comments. I have selected minor revision but really these are just minor 'tweaks' - I believe that limitations have been addressed apart from one aspect - see my comment below. I apologise for not identifying this in my first review.

E2 Comment re title: I have no problem with the first part as a question - I struggle with the second half of the title - suggest amending title to:

'Do you think medicines can be prescribed in a more eco-directed, greener way? Public and private focus groups' (opinions? attitudes and beliefs?) on the environmental impact of pharmaceuticals in the water environment in Scotland.'

E3: I think your revision of the Abstract is well done and I have no further changes to suggest.

MAIN MANUSCRIPT

Line 77: reference number [7] needs to be moved outside full stop.

Line 83: 'in' rather than is some locations.

Line 108: reference numbers 15,6 to be placed outside comma

Line 109: full stop missing - place before reference numbers 17-19

Line 112, 123: reference numbers should be outside commas

METHODS

I think that the methods are now very clear as to what was done and could be reproduced by other researchers - no further suggestions.

RESULTS -

I think Table 3 is an excellent suggestion by Dr Luis - thank you for adding as it it - it makes it easy to compare results across participant groups.

STRENGTHS AND LIMITATIONS

With regards to focus group prescriber recruitment, was it difficult to get prescribers to participate? Did it include prescribers who were ambivalent regarding EDSP? I suspect participants were those interested in sustainability and perhaps concerned for the environment - therefore, I suggest adding a sentence to acknowledge this potential bias but arguing that regardless of this potential response bias, the findings were still valuable to inform the next phase of the research study.

No other suggestions or amendments required.

VERSION 2 - AUTHOR RESPONSE

Reviewer: 3
Dr. Judith Singleton, Queensland University of Technology
Comments to the Author:
Dear authors,
Thank you for your comprehensive revision of the original manuscript and your comprehensive response to editorial and reviewers' comments. I have selected minor revision but really these are just minor 'tweaks' - I believe that limitations have been addressed apart from one aspect - see my comment below. I apologise for not identifying this in my first review.

- The authors again thank Reviewer 3 for their continued efforts in improving this manuscript!

R3.1 Comment re title: I have no problem with the first part as a question - I struggle with the second half of the title - suggest amending title to: 'Do you think medicines can be prescribed in a more eco-directed, greener way? Public and private focus groups' (opinions? attitudes and beliefs?) on the environmental impact of pharmaceuticals in the water environment in Scotland.'

- The title has been amended following the editor's suggestion. The authors hope this has addressed the reviewer's concerns.

R3.2 I think your revision of the Abstract is well done and I have no further changes to suggest.

- Thank you!

MAIN MANUSCRIPT

R3.3 Line 77: reference number [7] needs to be moved outside full stop.

- Amended – and addressed throughout where similar instances occur.

R3.4 Line 83: 'in' rather than is some locations.

- Amended

R3.5 Line 108: reference numbers 15,6 to be placed outside comma

- Amended – and addressed throughout where similar instances occur.

R3.6 Line 109: full stop missing - place before reference numbers 17-19

- Amended

R3.7 Line 112, 123: reference numbers should be outside commas

- Amended

METHODS
I think that the methods are now very clear as to what was done and could be reproduced by other researchers - no further suggestions.

- Thank you!

RESULTS -
I think Table 3 is an excellent suggestion by Dr Luis - thank you for adding as it it - it makes it easy to compare results across participant groups.

- The authors agree that the table is a great addition to the manuscript, and improves the readability and accessibility of results.

STRENGTHS AND LIMITATIONS
With regards to focus group prescriber recruitment, was it difficult to get prescribers to participate? Did it include prescribers who were ambivalent regarding EDSP? I suspect participants were those interested in sustainability and perhaps concerned for the environment - therefore, I suggest adding a sentence to acknowledge this potential bias but arguing that regardless of this potential response bias, the findings were still valuable to inform the next phase of the research study.

- The researchers found that recruiting prescribers was simpler than engaging with the public, likely as prescribers could be reached through professional networks (e.g., email, advertisement through work-based groups, etc) rather than relying on social media. Although some of the prescribers were aware of the issue – this appeared to be a new topic to several who participated in the focus groups (as highlighted in the results and discussion sections). However, the authors agree with the suggestion from the reviewer, and text was added to the Strengths and Limitations section to clarify these points (see lines 530-535).

No other suggestions or amendments required.

Reviewer: 3

If you have selected ‘Yes’ above, please provide details of any competing interests.: Not applicable
